# Recognition and management of acute kidney injury in children: The ISN 0by25 Global Snapshot study

**DOI:** 10.1371/journal.pone.0196586

**Published:** 2018-05-01

**Authors:** Etienne Macedo, Jorge Cerdá, Sangeeta Hingorani, Jiayi Hou, Arvind Bagga, Emmanuel Almeida Burdmann, Michael Rocco V., Ravindra Mehta L.

**Affiliations:** 1 University of California San Diego, Department of Medicine, Division of Nephrology, San Diego, California, United States of America; 2 Division of Nephrology, Department of Medicine, Albany Medical College, Albany, New York, United States of America; 3 Division of Nephrology, Department of Pediatrics, University of Washington, Seattle Children’s Hospital, Seattle, United States of America; 4 University of California San Diego, Altman Clinical and Translational Research Institute, San Diego California, United States of America; 5 Division of Pediatric Nephrology, All India Institute of Medical Sciences, New Delhi, India; 6 LIM 12, Division of Nephrology, University of Sao Paulo Medical School, Sao Paulo, Brazil; 7 Department of Internal Medicine, Section on Nephrology, Wake Forest School of Medicine, Winston-Salem, North Carolina, United States of America; University of Nottingham, UNITED KINGDOM

## Abstract

**Background:**

In low and middle-income countries, reliable data on the epidemiology of childhood acute kidney injury (AKI) is lacking. The Global Snapshot, conducted by the ISN “*0by25*” AKI initiative, was a world-wide cross-sectional, observational study to evaluate AKI in hospitalized patients. Here we report the pediatric results of this study.

**Patients and methods:**

We prospectively collected data on children who met the Kidney Disease Improving Global Outcomes AKI criteria during a 10-week window in late 2014. AKI risk factors, etiological factors, management and outcomes were recorded using standardized forms and protocols. Countries were classified according to their 2014 gross national income (GNI) per person into high-income countries (HIC), upper-middle income countries (UMIC) and low and low-middle income countries (LLMIC). Need for renal replacement therapy, mortality, and renal recovery were assessed 7 days after AKI diagnosis or at hospital discharge, whichever came first.

**Results:**

92 centers from 41 countries collected data on 354 pediatric AKI patients; 53% of the children developed AKI while hospitalized and 47% in the community. The most common etiological factors for AKI differed across GNI categories as well as between patients with community-acquired vs. hospital-acquired AKI. Children from HIC were younger, and larger proportion of AKI in this group were due to post-surgical complications vs. other etiologies when compared to other income categories. In patients with hypotension as the cause of AKI, the adjusted risk of death was almost 10-fold higher compared to patients without hypotension as an etiological factor for AKI development. Mortality was similar within AKI stages in HIC and UMIC. In LLMIC, patients with the highest AKI level of severity had higher mortality than patients in higher income categories. Patients from LLMIC and UMIC had a 57-fold and 11 fold higher adjusted risk of death, respectively, compared to patients from HIC.

**Conclusion:**

In resource-limited countries, pediatric AKI-associated mortality is disproportionately higher when compared to high-resource areas, especially among patients with more severe AKI.

## Introduction

In the developed world, the use of standard AKI definitions [1–3] has led to an improved understanding of the epidemiology and outcomes of this illness in both adult [4,5] and pediatric [6,7] populations; however, little data is available on the epidemiology of pediatric AKI in resource-limited regions of the world [8]. The scarcity of pediatric reports from these regions impairs the ability to improve AKI prevention and recognition; essential factors needed to decrease the incidence and improve the outcomes of pediatric AKI [8–15].

The “0by25” initiative of the International Society of Nephrology (ISN) has the ambitious goal of reaching zero preventable deaths from AKI across the world by 2025 [16]. The AKI Global Snapshot (GSN) [17] was an initial attempt to understand the incidence, causes, treatment and outcomes of AKI around the world, focusing on lower and lower-middle income group countries, which are underrepresented in the majority of AKI studies. In this report, we hypothesize that the causes and outcomes of pediatric AKI are dissimilar in diverse geographic and economic regions of the world.

## Patients and methods

The GSN study design has been described previously [17]. Briefly, this was a prospective observational study conducted worldwide via recruitment of health care volunteers across nephrology societies, meetings, a dedicated website (www.0by25.org), electronic communications and medical society membership. These physicians collected local AKI data, which was transmitted to a data-coordinating center utilizing a standardized electronic form. The University of California, San Diego, Human Research Protection Program approved this study. Participating providers obtained institutional review board approval from their institutions in accordance with local ethics regulations. Providers selected an index day and then screened suspected cases of AKI within a three-day window before or after the index day. Screened patients were followed to identify those that met pediatric KDIGO criteria for AKI. Detailed data was collected for eligible patients on the cause, treatment, and outcome of AKI until the time of hospital discharge, death or otherwise reaching the 7th day of follow-up (S1 Data Elements). Exclusion criteria included patients with chronic kidney disease (CKD) stage 5, known end-stage renal disease (ESRD) on dialysis, or with a functional renal transplant.

The most recent creatinine value prior to the development of AKI was considered the baseline creatinine level. If no baseline creatinine value was available, then the first creatinine level available during hospital admission was considered the reference creatinine value. We applied modified AKI KDIGO criteria [1,18,19] and defined *confirmed AKI* as an increase or decrease in serum creatinine of ≥0.3 mg/dl and/or an increase in creatinine of ≥ 50% from the reference value within a minimum of three days from the index day or a prior known baseline value. When urine volume was available, we considered *confirmed AKI* if urine output was <0.5 ml/kg/hour for six or more hours. We used the pRIFLE criteria to classify AKI severity [20] and the Schwartz formula [21] to estimate glomerular filtration rate (eGFR) in patients under 18 years of age. Urine output criterion is reported as urine volume adjusted by body weight over a 24-hour period. Countries were divided into High (HIC); Upper Middle (UMIC) and Low Middle and Low (LLMIC) income groups based on their 2014 gross national income (GNI) per person. Community-acquired AKI was defined as an episode of AKI when the initial event occurred outside of the hospital setting and the patient was subsequently admitted to the hospital with a diagnosis of AKI. Hospital acquired AKI was defined as an episode of AKI due to a kidney insult that occurred in hospitalized patients who developed de novo AKI during their hospital stay [22].

Outcomes included renal replacement therapy (RRT) requirement, renal recovery and mortality, and were assessed either at seven days or at last observation, if later than 7 days. When known, pre-AKI creatinine values and past medical history were used to determine if CKD was present at baseline. Renal recovery determination was based on the last available serum creatinine (SCr) and defined as “complete” if SCr was equal to or lower than at baseline or reference; “partial” if lower than the value at diagnosis but still higher than baseline or reference, and “no-recovery” if the SCr levels did not decrease and/or the patient remained on dialysis.

Data was entered into a secure web based platform (KEEP.distributedhealthlabs.org and keep.isn.org) that was accessible on the ISN 0 by 25 AKI website (www.0by25.org) [23]. We present continuous variables as mean (standard deviation, SD) or median (interquartile range, IQR), as appropriate. Normality was tested by the Kolmogorov—Smirnov test. The association between levels in each categorical covariate and three GNI levels was evaluated using the Chi- squared test. Normality was tested with the Kolmogorov—Smirnov test. Skewed distributions are described with medians and interquartile ranges and were compared with using the Kruskal—Wallis test.

Missing data for height were imputed using the 50^th^ percentile height based on age and sex from the 2006 WHO Multicenter Growth Reference Study child growth standards. (http://www.who.int/childgrowth/standards/technical_report/en/). Logistic regression was used to identify risk factors that may have contributed to mortality. In logistic regression, cases that are missing variables in the proposed model are dropped from the analysis, leaving only complete cases. For the model creation, GNI and age were fixed and every other risk factors were conditional on these two covariates. The likelihood ratio test (LRT) was used to compare the model with the additional risk factor to the previous model. We used a P value < 0.10 to indicate whether the additional risk factor was added. Analysis was conducted using SAS 9.4 (SAS Institute Inc., Cary, NC, USA) and R version 3.2.1.

## Results

**Fig 1:**
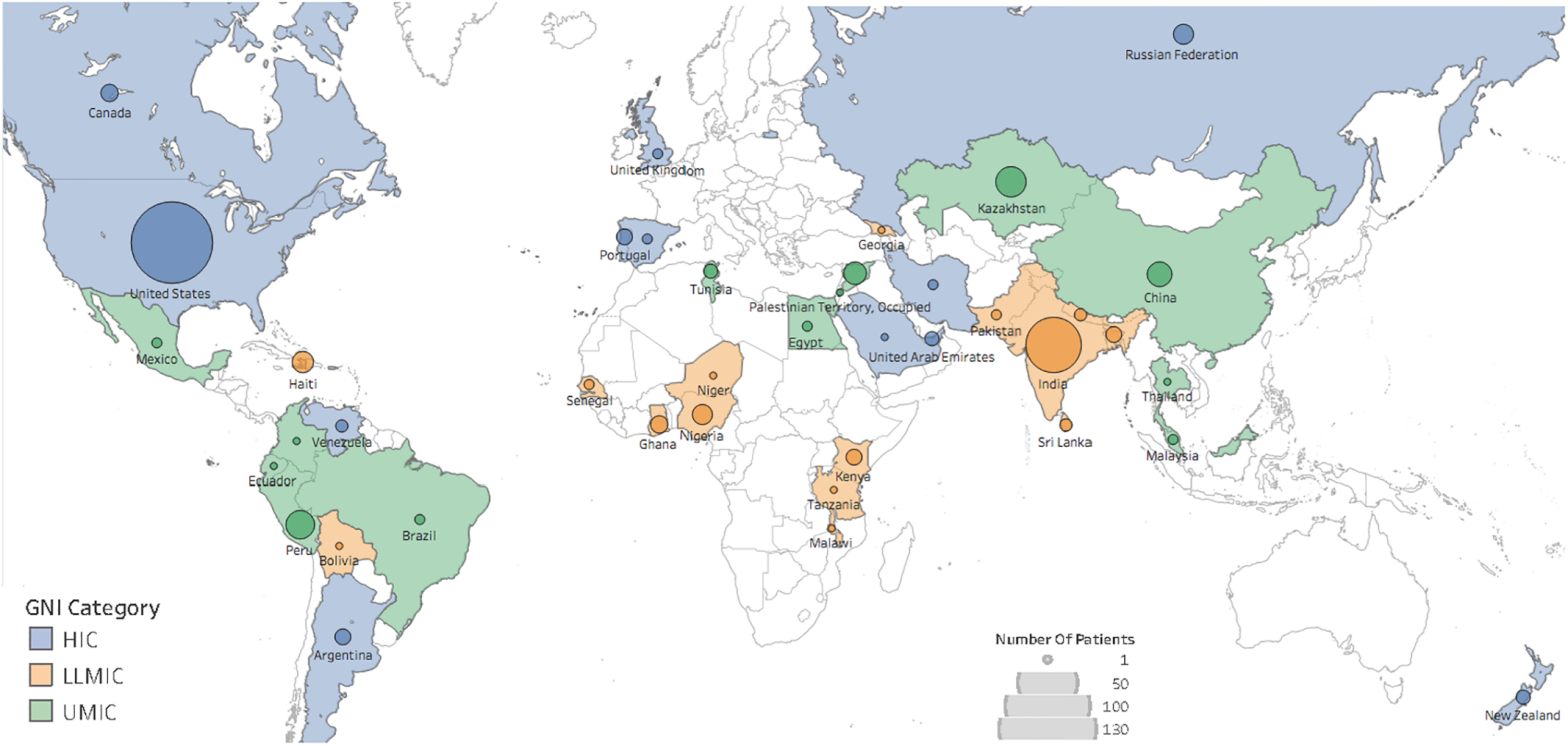
Patient distribution based on GNI category and geographical region. Color based on Gross national income, size of marks based on number of patients enrolled by country. HIC: high income country; UMIC: upper middle-income country; LLMIC: low and low middle income country. This graphic was made in Tableau Desktop.

Data on 354 pediatric patients with AKI were obtained from 92 providers from 41 countries. All geographic regions of the world provided patients to the survey (Fig 1). South Asia contributed the most patients (23, 25% of patients), followed by Africa (16, 17%), Latin America and the Caribbean (15, 16%), and North America (11, 12%). The majority of participating physicians were adult or pediatric nephrologists (87, 94.6%) and almost one -third of participating centers were in cities with a population of more than five million inhabitants (29, 32.6%). RRT was available in almost all (87, 97.8%) centers.

The median age of the patients was 2.0 (IQR 0.2–11.0) years; 87 (24%) adolescents (median 17y/o; IQR 15–17)), 123 (34%) were children (median 4y/o; IQR 2–7), 45 (13%) were infants (median 3.6 months; IQR 3–7), and 99 (28%) were neonates (median 1 week; IQR 0.2–4). Patients from HIC were younger (mostly neonates and infants) compared to UMIC and LLMIC (Table 1). While gender was evenly distributed in HIC, there was a clear male predominance in LLMIC (67%, p<0.005).

**Table 1:** Comorbidities, risk factors and location of AKI diagnosis in GNI country categories.

Variables	N	All	HIC	UMIC	LLMIC	*P*
			(n = 174)	(n = 72)	(n = 108)	
Age, years	354	2.0 (0.17, 11.0)	0.4 (0.03, 6.0)	8.0 (2, 16.5)	4.0 (0.3, 13.5)	<0.001
Boys	353	193 (54)	80 (46)	41 (57)	72 (67)	0.005
Community AKI	350	163 (46.0)	33 (19.0)	44 (61.1)	86 (79.6)	<0.001
**Location at diagnosis**						<0.001
Emergency		61(17.4)	10(5.8)	27(37.5)	24(22.6)	
Intensive care unit		136(38.9)	75(43.6)	25(34.7)	36(34.0)	
Outpatient		11(3.1)	2(1.2)	4(5.6)	5(4.7)	
Inpatient wards		142(40.6)	85(49.4)	16(22.2)	41(38.7)	
BMI, kg/m^2^	188	17.0 (14.3, 21.3)	17.8 (15.3, 21.3)	18.1 (15.8, 22.2)	15.0 (12.5, 18.3)	0.001
Chronic kidney impairment	354	26 (7.3)	11 (6.3)	6 (8.3)	9 (8.3)	0.77
Known baseline sCr		215	141 (80)	34 (47)	40 (37)	<0.001
Baseline sCr (micromol/l)		35.4(17.7, 69)	26.5(17.7, 44.2)	70.7(53.0, 94.0)	67.8(38.2, 114.9)	<0.001
eGFR ml/min/1.73 m2 (Schwartz equation)						
Adolescent (13–18 yr)	87	88.6 (68.4, 111.8)	112 (88.3, 125.1)	79.6 (68.1, 91.5)	84.2 (54.3, 87.5)	0.013
Child (>1–12 yr)	123	89.9 (47.2, 159.1)	146.2 (84.1, 187.3)	53.1 (38.7, 101.4)	59.5 (35.9, 87.8)	< .001
Infant (>4 wk-1yr)	45	74.8 (50.7, 112.3)	83.9 (51.2, 123.5)	78.4 (73.3, 104.1)	23.0 (8.2, 53.2)	0.026
Neonate (0–4 wks)	99	67.5 (40.5, 101.4)	67.6 (45.9, 101.4)	19.9 (19.2, 20.6)	NA (NA, NA)	0.029
Anemia (hemoglobin <9 g/dl)	354	58(16.4)	18(10.3)	14(19.4)	26(24.1)	0.008
**Risk factors**						
Dehydration	115	32.49	45 (26)	22 (30)	47 (43.5)	0.011
Hypotension, shock	113	31.92	46 (26)	31 (32)	43 (40)	0.043
Infection	104	29.38	33 (18.97)	23 (31.94)	48 (44.44)	<0.001
Nephrotoxic agents	71	20.06	23 (14)	14 (19)	33 (30)	0.3642
Primary kidney diseases	62	17.51	14 (8.05)	19 (26.39)	29 (26.85)	<0.001
Post-surgical	56	15.82	47 (27.01)	3 (4.17)	6 (5.56)	0.0218
Systemic diseases	48	13.56	23 (13.22)	16 (22.22)	9 (8.33)	<0.001
Cardiac diseases	41	11.58	34(19.54)	2(2.78)	5(4.63)	0.1334
Urinary obstruction	16	4.52	3(1.72)	4(5.56)	9(8.33)	0.0281

P value refers to difference among country categories (HIC, UMIC and LLMIC); HIC: high income country; UMIC: upper middle income country; LLMIC: low and lower middle income country. Values represent median (interquartile range) or number (proportion).

Overall, a similar number of patients developed AKI in the community (163, 46.0%) as in the hospital (187, 52.8%); however, when analyzed by GNI category, 34 (20%) of the identified AKI cases occurred in the community, compared with 87 (80%) in LLMIC (S1 Table). In HIC, approximately one-half of the patients (85, 49.4%) were diagnosed with AKI in the ICU or emergency department, whereas in LLMIC 41 (38.7%) were diagnosed in the ward or step-down unit (Table 1). Comorbid conditions were uncommon except for anemia, which was more frequent in LLMIC (24.1%) and UMIC (19.4%) compared to HIC (10.3%) (Table 1). Baseline SCr creatinine levels were higher in LLMIC (0.8 mg/dl; IQR 0.5–1.5 mg/dl) and UMIC (0.8 mg/dl; 0.6–1.1 mg/dl), compared to that found in HIC (0.3; 0.2–0.6 mg/dl) (Table 1).

There were differences in the etiology of AKI in HIC compared to UMIC and LLMIC. Chief factors associated with AKI in HIC were hypotension (30%), post-surgical complications (27%) and dehydration (26%). In contrast, dehydration was the most common etiologic factor in LLMIC (43.5%) and UMIC (30.6%) (Table 1). Infection, nephrotoxic medications and primary kidney diseases were more common AKI etiologies in LLMIC than in UMIC or HIC countries.

The causes of AKI also varied depending on the setting where AKI developed; in community-acquired AKI, the most frequent etiology was primary kidney disease (39, 24%), followed by hypotension/shock (29, 17%), dehydration (28, 17%) and systemic disease (23, 14%). Hypotension/shock (53, 27%) and post-surgical status complications were the most frequent etiologies in hospital- acquired AKI.

### AKI diagnosis and severity

The diagnosis of AKI was made exclusively by SCr criteria in 141 (39.8%); exclusively by urine output in 112 (31.6%); and by both criteria in 101(28.5%) patients. In HIC, SCr levels at diagnosis of AKI were lower than levels from other GNI categories. The first available estimated GFR during hospital admission was lower in UMIC and LLMIC patients than HIC patients (Table 2). Similarly, urine output was lower at diagnosis in LLMIC and UMIC patients compared to HIC patients. Most patients in LLMIC (94%), UMIC (85%) and HIC (73%) were diagnosed at the “Failure” (highest AKI) stage.

**Table 2:** Renal functions by GNI country category.

Variables	N	All	HIC	UMIC	LLMIC	*P*
			(n = 174)	(n = 72)	(n = 108)	
**At AKI diagnosis**						
SCr(micromole/l)	289	141.4 (61.0, 291.7)	77.28 (26.5, 167.7)	194.48 (119.3, 371.3)	177.90 (108.3, 401.5)	< .001
**eGFR ml/min/1.73 m2 (Schwartz equation)**						
Adolescent(13–18 yr)	87	27.3 (14.2, 41.9)	36.1 (21.4, 48.1)	27.3 (16.6, 36.9)	18.6 (11.8, 35.9)	0.033
Child(>1–12 yr)	123	19.48 (10.8, 44.3)	44.6 (18.9, 105.7)	17.8 (9.8, 31.5)	16.5 (8.0, 32.8)	< .001
Infant(>4 wks-1 yr)	45	26.3 (10.8, 44.0)	26.3 (16.5, 101.2)	20.6 (10.6, 35.7)	27.3 (7.6, 39.2)	0.005
Neonate (0–4 wks)	99	50.7 (14.8, 101.4)	67.6 (37.8, 101.4)	10.6 (9.9, 11.3)	12.7 (11.5, 14.0)	< .001
BUN (mg/dl)		16.0 (7.5, 30.3)	12.1 (5.5, 25.1)	20.0 (11.3, 33.1)	18.9 (7.5, 31.6)	< .001
Urine output past 24 h(ml/kg)		11.8 (4.5, 35.2)	20.8 (6.7, 39.9)	7.14 (3.5, 22.9)	10 (5, 37.5)	0.016*
**Criteria for AKI diagnosis**						
SCr (alone) (%)		141(39.8)	45(25.9)	35(48.6)	61(56.5)	<0.001
Oliguria (alone) (%)		112(31.6)	99(56.9)	2(2.8)	11(10.2)	0.77
SCr + UO (%)		101(28.5)	30(17.2)	35(48.6)	36(33.3)	<0.001
**Stage at diagnosis** pRIFLE						<0.001
Risk		115(39)	59(49)	17(23)	39(39)	
Injury		38(13)	21(17)	12(16)	5(5.1)	
Failure		137(47)	49(32)	43(59)	55(55)	

P value refers to difference among country categories (HIC, UMIC and LLMIC); HIC: high income country; UMIC: upper middle-income country; LLMIC: low and lower middle income country; SCr: serum creatinine. Values represent median (interquartile range) or number (proportion).

### Management and course

Procedures used to determine the etiology of AKI were urinalysis in 206 (59.0%), ultrasonography in 142 (40.7%) and renal biopsy in 20 (5.8%) patients. Urinalysis was performed more often in UMIC (62, 86.1%) and LLMIC (87, 82.9%) than in HIC (57, 33.1%). Ultrasonography was used more commonly in UMIC (42, 58.3%) and LLMIC (56, 53.3%) compared to HIC (44, 25.6%). Treatment of AKI consisted of intravenous fluids in 217 (62.2%), antibiotics in 188 (54.7%), diuretics in 112 (32.1%) and vasopressors in 70 (20.1%) patients.

As shown in Table 3, 74 (21.5%) patients received RRT; 66 (48%) of patients reached maximum stage Failure (S2 Table). Among the dialyzed patients, 18 were from HIC, 29 from UMIC, and 27 from LLMIC. Solute control was the primary indication for RRT, followed by fluid overload, and electrolyte or acid/base disturbances (Table 3). RRT modalities included peritoneal dialysis, intermittent hemodialysis, and continuous RR; the latter modality was not used in any LLMIC patient. Estimated GFR at the start of RRT was significantly lower in LLMIC patients than in those from the other two income categories (Table 2).

**Table 3:** Renal replacement treatment (RRT) characteristics, renal function at RRT initiation and at hospital discharge.

Variables	#patients	All (74)	HIC (18)	UMIC (29)	LLMIC (27)	P value
**At RRT initiation**						
SCr (micromole/l)	56	466.05 (204.00, 605.54)	196.00 (101.66, 376.58)	465.10 (327.08, 680.68)	530.40 (468.93, 614.38)	<0.001
eGFR ml/min/1.73 m2 (Schwartz equation)						
Adolescent(13–18 yr)	87	8.5 (7.6, 16.3)	12.4 (10.3, 14.4)	12.3 (8.0, 16.5)	6.90 (6.7, 7.3)	0.39
Child(>1–12 yr)	123	7.6 (6.8, 10.5)	12.9 (8.4, 14.6)	7.74 (5.2, 9.9)	7.49 (6.8, 8.5)	0.058
Infant(>4 wks-1yr)	45	25.4 (15.0, 39.6)	26.3 (24.5, 37.1)	10.0 (8.1, 28.5)	NA (NA, NA)	0.41
Neonate (0–4 wks)	99	16.1 (12.6, 19.3)	19.3 (17.7, 20.8)	NA (NA, NA)	9.0 (9.0, 9.0)	0.309
BUN	51	32.1 (21.8, 51.4)	32.0 (20.7, 46.0)	28.9 (19.8, 51.6)	35.7 (24.8, 51.4)	0.932
Urine output past 24h (ml/kg)	54	3.9(1.2, 11.4)	5.4(0.9, 40.3)	4.0(1.0, 8.1)	3.5(2.0, 8.6)	0.68
**Stage at RRT (pRIFLE)**						0.007
	Risk	6 (11)	5 (31)	0	1 (4.5%)	
	Failure	49 (89)	11 (68)	17 (100)	21 (95.5%)	
**Reasons for RRT initiation**						
Fluid overload		38(66.7)	12(70.6)	13(72.2)	13(59.1)	0.63
Solute control		48(84.2)	14(82.4)	16(88.9)	18(81.8)	0.82
Electrolyte or acid/base disturbance		38(66.7)	11(64.7)	9(50.0)	18(81.8)	0.1
**Dialysis modality**						
IHD		18(31.6)	1(5.9)	8(44.4)	9(40.9)	0.024
PD		27(47.4)	9(52.9)	6(33.3)	12(54.6)	0.35
CRRT		7(12.3	3(17.7)	4(22.2)	0	0.051
UF		3(5.3)	3(17.7)	0	0	0.023
SLED		1(1.8)	0	0	1(4.6)	1
**At last observation day**						
Cr SCr (micromole/l)	*126*	62.0(32.7, 132.6)	32.4(17.7, 59.0)	99.1(62.3, 210.5)	90.0(57.5, 176.8)	<0.0001
eGFR ml/min/1.73 m2 (Schwartz equation)						
Adolescent (13–18 yr)	87	50.7 (28.2, 92.7)	97.0 (47.9, 111.0)	50.5 (22.2, 78.8)	36.4 (25.2, 63.6)	< .001
Child (>1–12yr)	123	48.1 (25.4, 102.3)	107.0 (57.7, 166.5)	41.3 (18.4, 61.9)	33.1 (21.47, 57.8)	< .001
Infant (>4 wks-1yr)	45	56.9 (31.4, 101.3)	85.5 (50.3, 111.3)	36.0 (25.3, 46.9)	34.6 (24.5, 62.4)	< .001
Neonate (0–4 wks)	99	68.7 (38.6, 101.4)	68.7 (67.6, 102.2)	21.4 (20.2, 22.5)	21.6 (11.7, 33.4)	< .001
Stage at last observation (pRIFLE)						0.038
Risk		60(47.6)	34(63)	15(38.5)	11(33.3)	
Injury		14(11.1)	5(9.3)	5(12.8)	4(12.1)	
Failure		25(19.8)	7(13)	5(12.8)	13(39.4)	
On Dialysis		27(21.4)	8(14.8)	14(35.9)	5(15.2)	

P value refers to difference among country categories (HIC, UMIC and LLMIC); HIC: high income country; UMIC: upper middle-income country; LLMIC: low and lower middle income country; IHD: intermittent hemodialysis; UF: ultrafiltration; PD: peritoneal dialysis; CRRT: continuous renal replacement therapy; SLED: slow low efficient dialysis; SCr: serum creatinine. Values represent median (interquartile range) or number (proportion).

RRT was indicated but not begun in 7 (9.2%) of LLMIC, 6(14.0%) of UMIC and 2(1.3%) of HIC patients (*P* <0.0001). Futility was cited as the chief cause for withholding RRT in HIC, but in LLMIC, two patients were not dialyzed due to financial constraints and 3 due to lack of trained staff.

### Outcomes

#### Renal recovery

Renal recovery at seven days was complete in 128 (36.6%) patients and partial in 117 (33%) children. No renal recovery was seen in 58 (16%) patients; however, no data on renal recovery was available in 42 (12%) children. Non-recovery rates were significantly lower in HIC patients (12, 6.9%) than in UMIC (22, 30%) or LLMIC patients (24, 22%) (Table 4). Recovery rates from community-and hospital-acquired AKI were similar.

**Table 4:** Recovery status at seven days in patients receiving and not receiving renal replacement therapy by GNI category.

	Total		HIC		UMIC		LLMIC	
***Overall Recovery***								
Complete/Partial	245	69.2%	120a	69.0%	49a	68.1%	76a	70.4%
None	58	16.4%	12a	6.9%	22b	30.6%	24b	22.2%
Unknown	42	11.9%	39a	22.4%	1b	1.4%	2b	1.9%
***Need for dialysis***								
**Dialyzed**								
Complete	14	18.9%			5a	17.2%	9a	33.3%
Partial	26	35.1%	9a	50.0%	9a	31.0%	8a	29.6%
None	33	44.6%	9a	50.0%	14a	48.3%	10a	37.0%
Unknown	1	1.4%			1a	3.4%		
**Non-dialyzed**								
Complete	114	42.1%	63a	41.2%	16a	37.2%	35a	46.7%
Partial	91	33.6%	48a	31.4%	19a	44.2%	24a	32.0%
None	25	9.2%	3a	2.0%	8b	18.6%	14b	18.7%
Unknown	41	15.1%	39a	25.5%			2b	2.7%
***Maximum AKI Stage***								
**Risk**								
Complete	69	40.1%	45a	40.2%	5a	31.3%	19a	43.2%
Partial	45	26.2%	22a	19.6%	9b	56.3%	14a,b	31.8%
None	12	7.0%	4a	3.6%	2a	12.5%	6a	13.6%
Unknown	39	22.7%	38a	33.9%			1b	2.3%
**Injury**								
Complete	21	47.7%	10a	43.5%	7a	58.3%	4a	44.4%
Partial	21	47.7%	12a	52.2%	4a	33.3%	5a	55.6%
None	2	4.5%	1a	4.3%	1a	8.3%		
**Failure**								
Complete	38	27.5%	8a	20.5%	9a	20.5%	21a	38.2%
Partial	51	37.0%	23a	59.0%	15a,b	34.1%	13b	23.6%
None	44	31.9%	7a	17.9%	19b	43.2%	18a,b	32.7%
Unknown	3	2.2%	1a	2.6%	1a	2.3%	1a	1.8%

HIC: high income country; UMIC: upper middle-income country; LLMIC: low and lower middle income country. Values represent number (column proportion). Values in the same row and sub table not sharing the same subscript are significantly different at p< .05 in the two-sided test of equality for column proportions. Cells with no subscript are not included in the test. Tests assume equal variances.

Complete renal recovery was not different among patients reaching maximum “Risk” or “Injury” levels of AKI severity. Non-recovery was more frequent in “Failure” patients from UMIC and LLMIC. Of dialyzed patients, 33 (44%) did not recover renal function by the day of last observation. Patients not requiring RRT recovered function more frequently than those requiring RRT (205, 75.6% versus 33, 44%, respectively; P <0.001) (Table 4).

In the univariate analysis, non-recovery (complete or partial) was associated with hypotension and systemic disease, oliguria at diagnosis and higher baseline SCr.

#### Mortality

**Fig 2:**
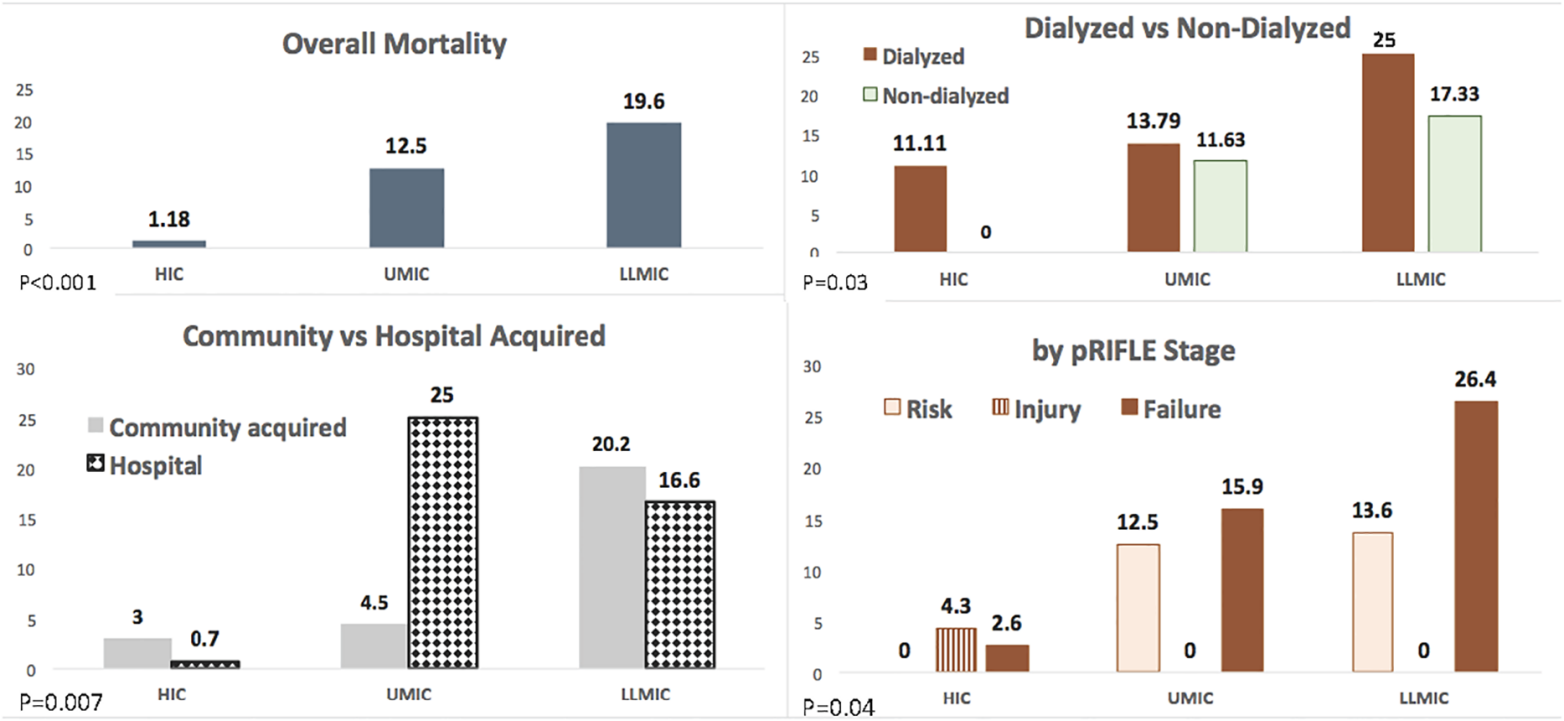
Mortality frequency in the overall cohort, and by AKI development location (community vs hospital acquired AKI), by need of ICU and renal replacement therapy. HIC: high income country; UMIC: upper middle-income country; LLMIC: low and lower middle income country. P values refer to difference within the GNI groups.

Overall, 314 (91%) patients were alive seven days after AKI diagnosis (Fig 2). Mortality frequency ranged from 1.2% in HIC patients to 19.6% in LLMIC patients. After adjusting for post-operative AKI alone, the increased risk persisted in LLMIC (OR 17; 95% CI 4.9–114) and UMIC (OR 10.2; 95% CI 2.5–69) patients.

In UMIC patients, hospital mortality was significantly higher than community acquired AKI, but was not different in HIC and LLMIC patients (Fig 2). In both ICU and non-ICU settings, more deaths occurred in LLMIC patients (39.4% and 10.1% respectively) than in UMIC (8, 32% and 1, 2.1%) and HIC patients (2, 2.85% and 0%; P <0.0001). Mortality in patients receiving RRT was higher across all GNI categories, and reached 25% in LLMIC, compared to 10% in HIC and 13.7% in UMIC (p = 0.03) (Fig 2). No significant differences were found in mortality frequency by maximum AKI stage in HIC and UMIC patients. In LLMIC, the mortality was higher in patients reaching maximum “Failure” stage.

In the univariate analysis, risk factors associated with mortality included lower baseline eGFR, hypotension and GNI category. Pre-existing chronic heart disease, cardiac factors causing AKI and primary kidney diseases were associated with a lower risk for mortality. In multivariable logistic analysis, only GNI category and hypotension remained significantly associated with mortality (Table 5). The adjusted risk of death among patients from LLMIC was 57-fold higher than in HIC patients, and 11-fold higher in patients from UMIC compared to HIC patients. In patients with hypotension as the cause of AKI, the adjusted risk of death was almost 10-fold higher compared to patients without hypotension as an etiological factor for AKI development (Table 5).

**Table 5:** Multivariable logistic regression for mortality in patients with AKI.

	Estimate	Odds Ratio	95% CI
**(intercept)**	-6.56	-	
UMIC*	2.43	11.45	1.02–318.63
LLMIC*	4.06	57.92	6.65–1616.44
**Chronic heart disease**	-0.48	0.62	0.007–21.17
**Baseline eGFR**	0.01	1.01	0.99–1.02
**Hypotension**	2.29	9.92	2.11–57.44
**Cardiac causes**	-0.28	0.75	0.01–33.11
**Primary kidney diseases**^#^	-0.76	0.46	0.02–3.58

* HIC as reference;

^#^ include glomerulonephritis, vasculitis, AIN, pyelonephritis.

UMIC: upper middle income countries; LLMIC: low and low middle income countries.

## Discussion

The Global Snapshot is the first worldwide prospective cross-sectional study of AKI resulting in hospitalization that was designed to capture data from different health care settings across six continents. A prior publication provided data for all participants in the GSN [23]; this article focuses on the pediatric patients represented in this study. The provenance of this pediatric cohort is mostly from North America and South Asia, but all other geographic areas were represented including Africa, an area where little information was heretofore available [24](Fig 1). In our sample, patients were mainly diagnosed in large urban centers and managed by nephrologists, which is in stark contrast to the prevalent situation in LLMIC, where nephrologists are scarce or absent, and where AKI occurs in the community and rarely reaches large urban centers [13,14,16,17,24–26]. A unique aspect of our sample was that almost half of the patients were considered to have developed AKI in the community setting.

Acute kidney injury occurred in different circumstances in HIC compared to UMIC and LLMIC. Patients from HIC were younger (predominantly neonates and infants), their baseline SCr was more readily available, and their AKI was predominantly attributed to post-operative cardiac complication. Conversely, in LLMIC, AKI commonly developed in the community and baseline renal function data was less frequently available. In LLMIC, the high proportion of community-acquired AKI is likely associated with public health infrastructure-related problems such as poor sanitation, endemic infections and waterborne gastroenteritis, suggesting that primary prevention, early risk assessment, and training of first-contact care providers may decrease the incidence and improve the outcomes of these preventable AKI cases [13,14,16,26].

As expected in this young cohort, the frequency of comorbid conditions was low across the GNI categories except for anemia, which was present in 24% of the patients in LLMIC. The anemia seen in LLMIC patients may be a marker for prevalent malnutrition, chronic infection and/or endemic malarial and parasitic disease, which in turn might impact the incidence and outcome of AKI in this population [27–33].

Age was not associated with mortality, suggesting that settings and etiologies might be stronger determinants of the dramatic differences in outcome when stratified by GNI. Interestingly, the LLMIC cohort showed a sizable number of patients with primary kidney diseases such as acute glomerulonephritis, illustrating differing characteristics and etiologies of AKI in the developing world; this observation has been reported in other developing country cohorts [24,34,35]. Conversely, a more frequent diagnosis of primary kidney diseases may be the result of indication bias, as renal biopsies were more commonly done in UMIC and LLMIC than in HIC.

Urine output, an important warning sign of impending kidney dysfunction [36], was available in only half of the patients on the day of AKI diagnosis. The high prevalence of neonates, in whom accurate urine output quantification is challenging, may be part of the reason for this lack of data. When available, urine output volume adjusted by weight was significantly lower at diagnosis in LLMIC and UMIC compared to HIC. The importance of urine output assessment in children has been recently demonstrated in an epidemiologic study involving more than 32 ICUs worldwide [15]. In this study, the use of SCr alone would have resulted in missing 67% of AKI cases. Our multivariable analysis for kidney recovery emphasizes the importance of urine output assessment, and also demonstrated that patients with both oliguria and elevated serum creatinine levels had more severe AKI and were less likely to recover renal function completely [36,37].

In the overall GSN cohort, a similar proportion of children and adults (80%) from low and low middle-income countries developed AKI in the community [17]. The etiological factors leading to AKI development were similar: mainly dehydration and hypotension. However, in LLMIC, almost all children were diagnosed at the most severe pRIFLE stage (Failure), as compared with 58% of adults who were diagnosed at the equivalent KDIGO Stage 3 [17]. These differences in time to recognition as well as the increased mortality rate among children demonstrate the urgent need to improve timely access to care centers, and to identify the systematic problems causing such delays [17]. Commonly missed or delayed AKI recognition reflects a lack of awareness of the possibility of kidney disease as well as a lack of resources for diagnosis and management [14,26,38]. In these resource-limited regions, where serum creatinine tests are often unavailable or unaffordable, a lack of appreciation of the severity of the kidney injury likely leads to late referrals to higher levels of care. The higher frequency of community acquired AKI in LLMIC raises the concern that in those limited-resource areas, an even larger number of community cases may have never reached the urban centers and likely remain unaccounted for [14,16,24,35]. Education directed at increasing the awareness of the impact of AKI, the provision of point of care tests, the development and implementation of regionally appropriate kidney care guidelines and the facilitation of prompt referral should all contribute to improvements in the recognition, diagnosis, initial management and monitoring of kidney function [8,14,22,26].

Although by GSN design, patient follow-up was short (seven days), in that limited time window, in LLMIC we were able to demonstrate a much higher mortality rate associated with community- acquired AKI than that observed in HIC. This important finding again reflects the vulnerability of pediatric AKI patients in those regions of the world (Fig 2). Multiple reasons likely contributed to the higher mortality rate, including severe infections, late presentation, delayed recognition and diagnosis, and delayed [14], incomplete or unavailable RRT, all of which have been shown to lead to poor outcomes in developing countries [15–17,24,25,35,39–43].

Many LLMIC countries fail to offer RRT and financial constraints limit the duration of treatment, leading to severely increased mortality. In those countries, improving AKI awareness and promoting risk stratification, earlier diagnosis and treatment should significantly improve outcomes and spare children from the need for more severe, costlier and commonly unaffordable treatments, and from death [22,40, 44,45].

### Strengths and limitations

The GSN pediatric snapshot is a survey of a convenience sample that may over-represent certain demographic and socioeconomic factors. Firstly, more than 50% of the patients reported were from North America or South Asia. Secondly, urban areas were more likely to report AKI cases than rural areas. Thirdly, heterogeneous age ranges in different country income categories limit our understanding of the overall picture of AKI in these settings. Given that most of the data in GSN was reported by nephrologists, and the actual scarcity or absence of nephrologists in LLMIC, it is very likely that less severe cases of AKI, seen in the community and treated by non-specialists, were not included in the sample.

Outcomes were assessed at seven days or at hospital discharge. This time frame is not long enough to determine the full impact of AKI in mortality or the overall recovery from AKI. In LLMIC, true baseline SCr values were infrequently available. These limitations make it difficult to accurately assess the apparent severity of AKI on presentation, and complicates the distinction between late AKI and AKI superimposed on CKD. On the other hand, the better recovery demonstrated among LLMIC children suggests that the higher serum creatinine levels at hospital admission may actually be associated with a late presentation (as demonstrated by others) [14], again emphasizing the importance of early AKI recognition in achieving better outcomes [22]. This problem requires study in the field and in predominantly rural communities; such studies are being currently conducted in Bolivia, Mali and Nepal, as the International Society of Nephrology “0 by 25” Pilot Study [46].

Notwithstanding these limitations, this snapshot provides data from areas in low and middle-income regions of the world, that has rarely been reported before. The GSN was designed to track patients before AKI confirmation, thus providing an opportunity to ascertain early AKI etiology. Our results shows that pediatric AKI-associated mortality is disproportionately higher in low resource areas when compared to high-resource areas. These results also confirm our previous findings [16,47] that better investment in education and public health system infrastructure, including human resources, have the potential to significantly improve outcomes in children with AKI. We deeply hope that the evidence provided by this report will generate actionable items, and inform the design of current “0by25” pilot projects that aim to determine pathways to reduce the currently dramatic pediatric AKI morbidity and mortality around the world.

## Supporting information

S1 Acknowledgments Alphabetical list of participating centers by region & country. (DOCX)

S1 Data Elements (DOCX)

S1 Table Main patient characteristics, risk factors and outcomes by development location of AKI. (DOCX)

S2 Table Development location and outcomes by maximum AKI stage during observation period. (DOCX)
