# Supplementary material for: Recognition and management of acute kidney injury in children: The ISN 0by25 Global Snapshot study
Source: PLoS One. 2018 May 1;13(5):e0196586. doi: 10.1371/journal.pone.0196586 (PMC5929512; doi:10.1371/journal.pone.0196586)
Supplement: S1 Acknowledgments — (DOCX) [file pone.0196586.s001.docx]

**S1 Acknowledgments**

*Alphabetical list of participating centers by region & country*

1. **Africa**

**Egypt:** Tanta University Hospital (S Elattar; K Okasha)

**Ghana:** Komfo Anokye Teaching Hospital (S Antwi)

**Kenya:** Mater Hospital (G Moturi); Mombasa Hospital (M Sood); PGH Nakuru (J Kiyiapi)

**Malawi:** Queen Elizabeth Hospital (R Evans)

**Niger:** Hopital National (H Moussa Diongole)

**Nigeria:** Ahmadu Bello University Teaching Hospital (I Bosan ); JOS University Teaching Hospital (Z Mark Gimba); Lagos State University Teaching Hospital (T Umeizudike); University of Calabar Teaching Hospital (EEffa); University of Port Harcourt Teaching Hospital (FEke);

**Senegal:** Aristide Le Dantec University Hopital (A Lemrabott); Centre Hospitalier Regional de Saint-louis (D Ibrahima Mbemba)

**Tanzania:** Muhimbili National Hospital (J Shoo)

**Tunisia:** Hedi Chaker Hospital (K Kammoun); University Hospital of Sahloul (S Mabrouk)

**Latin America & The Caribbean**

**Argentina:** Hospital De Niños Pedro De Elizalde (S Martin); Hospital Universitario Austral (G Fragale)

**Bolivia:** Caja Nacional de Salud (R Claure-Del Granado; G Zeballos)

**Brazil:** Instituto do Cancer -ICESP - Faculdade de Medicina da USP (E Burdmann); Hospital Estadual de Bauru (D GeraAbrão)

**Colombia**: Hospital Militar Central (J Echeverri)

**Ecuador:** Hospital Enrique Garces (D Jimenez)

**Haiti:** HUEH (J Exantus)

**Mexico:** Hospital Infantil de Tlaxcala (M Cruz Angulo)

**Peru:** Hospital Honorio Delgado Espinoza (J Hinostroza Yanahuaya); Hospital Nacional Dos Mayo (J Hernandez); Servicio De Nefrologia Del Hospital Nacional Cayetano Heredia (C Loza Munarriz); Instituto Nacional de Salud del Nino (M Cisneros Mallcco)

**Uruguay:** Servicio M Dico Integral. Montevideo (R Lombardi)

**Venezuela:** Hospital Raul Leoni (N Pernalete); Hospital Dr. Manuel Nunez Tovar (G Velasquez)

1. **Middle East**

**Iran, Islamic Republic of**: Imamreza Teachinig Hospital (M Ardalan)

**Palestinian Territory, Occupied:** Hebron Governmental Hospital (M Qawasmeh)

**Saudi Arabia**: King Abdulaziz Medical City, Jeddah (M Qureshi)

**Syrian Arab Republic**: University Children Hospital in Damascus (H Wannous)

**United Arab Emirates**: Sheikh Khalifa Medical City (G Kumar)

1. **North & East Asia**

**China:** State Key Laboratory of Kidney Diseases, Department of Nephrology, Chinese PLA General Hospital (Z Feng); Jinan (S Sun); Renji Hospital (J Wong; S Jin; D Li;W Zhou); Ruijin Hospital (X Li; H Ren; W Zhang; A Chang; J Ying); Xinhua Hospital (W Niu)

**Taiwan, Province of China**: National Taiwan University hospital (H Pan)

1. **North America**

**Canada**: BC Children's Hospital (C Mammen)

**United States**: Children's Hospital Colorado (D Sorann); Children's Hospital of Alabama (L Dill); Children’s Hospital Colorado (KGist); Cincinnati Children's Hospital Medical Center (S Goldstein); Joe DiMaggio Children's Hospital (A Constantinescu); Rady Children's Hospital (L Nguyen); Seatlle Children's Hospital (S Hingorani); Texas Children’s Hospital (A Arikan); University of California, San Diego (C Cepeda)

1. **Oceania & South East Asia**

**Malaysia:** Hospital Selayang (H Wong); Hospital University Sains Malaysia (T Hussain)

**New Zealand**: Starship Children's Hospital (T Kara)

**Thailand:** King Chulalongkorn Memorial Hospital (N Srisawat)

1. **Russia & CIS**

**Georgia:** High Technology Medical Center, Center for development of Nephrology, University Clinic (I Tchokhonelidze)

**Kazakhstan:** National Research Center for Maternal and Child Health (M Khvan)

**Russian Federation:** Child City Clinical Hospital (A Eremeeva); Orenburg Regional Children's Hospital (M Kagan); Rogachev Federal Research Center for Pediatric Hematology, Oncology and Immunology (M Aksenova)

1. **South Asia**

**Bangladesh:** BSMMU (M Alam); Kidney Foundation; Hospital & Research Institute, Dhaka. (M Arefin); North East Medical College Hospital (M Islam); Shaheed Suhrawardy Medical College (R das Gupta)

**India:** Chirayu Hospital (M Doshetty); Department of Pediatrics, Jawaharlal Nehru Medical College, Aligarh Muslim University, Aligarh, Uttar Pradesh ( K Afzal ); Institute of Child Health (R Sinha); Kem Hospital Pune (J Sharma); Madras Medical College (G Natarajan); Osmania Medical College and Hospital (M Sahay); SRM Medical College Hospital (P Raghavan); Seth GS Medical College and KEM Hospital, Parel, Mumbai (T Jamale); Meenakshi Mission Hospital and Research Centre (K Sampathkumar):

**Nepal:** BP Koirala Institute of Health Sciences (S Sharma); Manipal Teaching Hospital (K Paudel); Patan Hospital (B Pandey)

**Pakistan:** Doctors Hospital And Medical Center (A Mansur); Shaikh Zayad Hospital (S Acharya)

**Sri Lanka:** Sirimavo Bandaranayake Specialized Children's Hospital (I Perera)

1. **Western Europe**

**Portugal:** CHSJoao (A Teixeira)

**Spain:** Hospital Universitario Virgen de la Arrixaca (J Ros, R Rodado); Hospital Universitario de Canarias (E Porrini)

**United Kingdom:** Alder Hey Children's NHS Foundation Trust Hospital (L Oni)

*International Society of Nephrology 0by25 Operations Group*

John Feehally, Fredric Finkelstein, Guillermo García-García, Vivekanand Jha, Norbert H Lameire, Nathan W Levin, Andrew Lewington, Raúl Lombardi, Etienne Macedo, Eliah Aronoff-Spencer, Marcello Tonelli, and Giuseppe Remuzzi
