# Supplementary material for: Recognition and management of acute kidney injury in children: The ISN 0by25 Global Snapshot study
Source: PLoS One. 2018 May 1;13(5):e0196586. doi: 10.1371/journal.pone.0196586 (PMC5929512; doi:10.1371/journal.pone.0196586)
Supplement: S1 Data Elements — (DOCX) [file pone.0196586.s002.docx]

**S1 Data Elements**

The “Patient Information” questionnaire, acquired data on patient demographics (age, gender, ethnicity); comorbidities; and AKI susceptibilities (dehydration, volume depletion, advanced age, gender, race, ethnicity, chronic kidney disease, chronic liver, heart, and lung disease, diabetes, cancer, anemia, and environmental risks). In the “Initial Data” section, we collected data on patient location and exposures associated with the development of AKI (sepsis, critical illness, shock, burns, trauma, cardiac and major non-cardiac surgery, nephrotoxic drugs, contrast agents, poisonous plants and animals). The “Diagnosis and Treatment” form, captured on how AKI was diagnosed (sCr, urine dipstick, urinalysis, labs, ultrasound, biopsy, other imaging studies) and confirmed. In addition, information on treatment and available resources, non-dialytic treatment (amount and type of fluids, medications, etc.), and renal support (indication, modality, frequency, duration) were recorded. Providers also completed the “Outcomes” forms 7 days after AKI confirmation or at last observation if earlier. Assessed outcomes included mortality, cause of death, level of renal recovery, and disposition at 7 days and at hospital discharge and scheduled physician follow up.
