# Supplementary material for: Recognition and management of acute kidney injury in children: The ISN 0by25 Global Snapshot study
Source: PLoS One. 2018 May 1;13(5):e0196586. doi: 10.1371/journal.pone.0196586 (PMC5929512; doi:10.1371/journal.pone.0196586)
Supplement: S1 Table — (DOCX) [file pone.0196586.s003.docx]

**S1 Table** – Main patient characteristics, risk factors and outcomes by development location of AKI.

|  | Hospital Acquired | | Community Acquired | |
| --- | --- | --- | --- | --- |
| GNI | | | | |
| HIC | 141_a_ | 73.8% | 33_b_ | 20.2% |
| UMIC | 28_a_ | 14.7% | 44_b_ | 27.0% |
| LLMIC | 20_a_ | 10% | 86_b_ | 52.8% |
| Age group |  |  |  |  |
| Neonate (0-4wks) | 87_a_ | 45.5% | 12_b_ | 7.4% |
| Infant(>4wks-1yr) | 27_a_ | 14.1% | 18_a_ | 11.0% |
| Child(>1-12yr) | 45_a_ | 23.6% | 78_b_ | 47.9% |
| Adolescent(13-18) | 32_a_ | 16.8% | 55_b_ | 33.7% |
| Diabetes mellitus | 2_a_ | 1.0% | 4_a_ | 2.5% |
| Chronic liver disease | 4_a_ | 2.1% | 1_a_ | 0.6% |
| Chronic heart disease | 7_a_ | 3.7% | 4_a_ | 2.5% |
| Chronic kidney impairment | 8_a_ | 4.2% | 18_b_ | 11.0% |
| Most important etiological factor | | | | |
| Dehydration | 24_a_ | 12.6% | 28_a_ | 17.2% |
| Hypotension and shock | 53_a_ | 27.7% | 29_b_ | 17.8% |
| Cardiac | 20_a_ | 10.5% | 3_b_ | 1.8% |
| Liver | 2_a_ | 1.0% | 3_a_ | 1.8% |
| Primary kidney diseases | 6_a_ | 3.1% | 39_b_ | 23.9% |
| Urinary obstruction | 3_a_ | 1.6% | 7_a_ | 4.3% |
| Infections | 15_a_ | 7.9% | 15_a_ | 9.2% |
| Pregnancy related | 1_a_ | 0.5% | 0^1^ | 0.0% |
| Systemic diseases | 13_a_ | 6.8% | 23_b_ | 14.1% |
| Nephrotoxic agents | 7_a_ | 3.7% | 11_a_ | 6.7% |
| Poisoning | 1_a_ | 0.5% | 2_a_ | 1.2% |
| Post-surgery | 42_a_ | 22.0% | 2_b_ | 1.2% |
| Outcomes | | | | |
| Need for dialysis | 19_a_ | 9.9% | 38_b_ | 23.3% |
| Mortality | 11_a_ | 5.9% | 20_b_ | 12.3% |
| Renal recovery | | | | |
| complete | 72_a_ | 38.5% | 56_a_ | 34.4% |
| none | 19_b_ | 10.2% | 39_a_ | 23.9% |
| partial | 53_b_ | 28.3% | 64_a_ | 39.3% |

HIC: high income country; UMIC: upper middle income country; LLMIC: low and lower middle income country. Values represent number (proportion). Note: Values in the same row and subtable not sharing the same subscript are significantly different at p< .05 in the two-sided test of equality for column proportions. Cells with no subscript are not included in the test. Tests assume equal variances.
