# Supplementary material for: Recognition and management of acute kidney injury in children: The ISN 0by25 Global Snapshot study
Source: PLoS One. 2018 May 1;13(5):e0196586. doi: 10.1371/journal.pone.0196586 (PMC5929512; doi:10.1371/journal.pone.0196586)
Supplement: S2 Table — (DOCX) [file pone.0196586.s004.docx]

**S2 Table** – Development location and outcomes by maximum AKI stage during observation period**.**

|  |  | Risk | | Injury | | Failure | |
| --- | --- | --- | --- | --- | --- | --- | --- |
| AKI Development location | community | 49_a_ | 43.0% | 15_a_ | 39.5% | 92_b_ | 66.7% |
|  | hospital | 65_a_ | 57.0% | 23_a_ | 60.5% | 46_b_ | 33.3% |
| Patient location | emergency | 19_a_ | 16.7% | 11_a_ | 28.9% | 31_a_ | 22.5% |
|  | ICU | 46_a_ | 40.4% | 15_a_ | 39.5% | 56_a_ | 40.6% |
|  | outpatient | 4_a_ | 3.5% | 1_a_ | 2.6% | 6_a_ | 4.3% |
|  | ward | 45_a_ | 39.5% | 11_a_ | 28.9% | 45_a_ | 32.6% |
| Need for dialysis |  | 4_a_ | 4% | 2_a_ | 5% | 50_b_ | 36% |
| Mortality |  | 5_a_ | 4% | 1_a,b_ | 3% | 22_b_ | 16% |
| Recovery status |  |  |  |  |  |  |  |
|  | complete | 53_a_ | 46.5% | 19_a_ | 50.0% | 38_b_ | 27.5% |
|  | none | 9_a_ | 7.9% | 2_a_ | 5.3% | 44_b_ | 31.9% |
|  | partial | 40_a_ | 35.1% | 17_a_ | 44.7% | 51_a_ | 37.0% |
|  | unknown | 10_a_ | 8.8% | 0^1^ | 0.0% | 3_b_ | 2.2% |

Note: Values in the same row and subtable not sharing the same subscript are significantly different at p< .05 in the two-sided test of equality for column proportions. Cells with no subscript are not included in the test. Tests assume equal variances.
